# Systematic Review Evaluating the Effects of Glucagon-Like Peptide-1 (GLP-1) Receptor Agonist Use on the Postoperative Outcomes of Patients Undergoing Arthroplasty Procedures

**DOI:** 10.7759/cureus.105269

**Published:** 2026-03-15

**Authors:** Hannah Tabor, Fenu Ediripolage, Nimra Akram, Irrum Afzal, Sarkhell Radha

**Affiliations:** 1 Orthopaedics, City St George's, University of London, London, GBR; 2 Orthopaedics, South West London Elective Orthopaedic Centre, Epsom, GBR; 3 Orthopaedics, Croydon University Hospital, London, GBR

**Keywords:** complication, glp-1 receptor agonists, knee arthroplasty, postoperative outcome, total hip arthroplasty (tha), total joint arthroplasty, total shoulder arthroplasty

## Abstract

Glucagon-like peptide-1 receptor agonists (GLP-1 RAs) are increasingly used for weight loss and may therefore have a role in preoperative optimisation for arthroplasty candidates. However, their effect on arthroplasty outcomes remains unclear. This systematic review aims to evaluate existing evidence on preoperative GLP-1 RA use and postoperative outcomes following joint arthroplasty, and to identify priorities for future research and perioperative optimisation. A systematic literature search of PubMed, Embase, ClinicalTrials.gov and the Cochrane Library was conducted for articles published from inception to 29th July 2025, using terms related to GLP-1 RAs and arthroplasty, in accordance with the PRISMA 2020 guidelines. Studies were included if they assessed postoperative outcomes in adult patients undergoing arthroplasty with documented preoperative GLP-1 RA use. Data were extracted on study design, participant characteristics, GLP-1 RA use, arthroplasty procedure, outcome measures, duration of follow-up, and key results. Fifteen retrospective studies met the inclusion criteria, comprising a total of 39,355 patients undergoing hip, knee or shoulder arthroplasty. Nine studies reported more favourable postoperative outcomes among GLP-1 RA users, three showed mixed results, two predominantly reported worse outcomes, and one found no significant difference. The most frequently reported favourable associations were lower periprosthetic joint infection and hospital readmission rates following hip and knee arthroplasty, particularly among individuals with diabetes or morbid obesity, although results were not uniform across studies. In contrast, evidence relating to shoulder arthroplasty outcomes was limited and showed greater variability. Preoperative GLP-1 RA use has been associated with lower rates of periprosthetic joint infection and readmission in several retrospective database studies, particularly in diabetic and morbidly obese arthroplasty populations; however, the overall certainty of evidence is low due to non-randomised designs, heterogeneity and risk of bias. These findings should be considered hypothesis-generating, and well-designed prospective studies and randomised controlled trials are required to establish the role of GLP-1 RAs in preoperative optimisation.

## Introduction and background

Obesity is increasing in prevalence worldwide, having more than doubled in adults since 1990 [1], and is associated with significant multisystem health consequences [2,3]. In England, 28% of adults were classified as obese and a further 36% as overweight in 2022 [4]. Obesity is an established risk factor for osteoarthritis, particularly in weight-bearing joints, and contributes to earlier progression to end-stage disease requiring total joint arthroplasty [5]. It also predisposes patients to poorer surgical outcomes, including periprosthetic joint infection (PJI), impaired wound healing, prolonged hospitalisation, longer operative times and higher revision rates [5,6].

As such, obesity represents a growing challenge for orthopaedic surgeons. Obesity is closely linked to type 2 diabetes, hypertension, cardiovascular disease, immune dysregulation, and respiratory compromise, all of which contribute to perioperative morbidity and mortality. Consequently, clinical guidelines emphasise the importance of preoperative weight optimisation for elective surgery [7,8].

Glucagon-like peptide-1 receptor agonists (GLP-1 RAs) have emerged as effective pharmacological options for weight reduction. These agents reduce appetite, delay gastric emptying and enhance insulin secretion [9]. Initially developed for glycaemic control in type 2 diabetes, GLP-1 RAs have demonstrated their significant efficacy for weight loss in multiple randomised controlled trials [10-12]. In the UK, semaglutide (Wegovy), tirzepatide (Mounjaro) and some brands of liraglutide are now licensed for weight management alongside lifestyle modifications in individuals with obesity or those overweight with weight-related health problems [13].

The uptake of GLP-1 RAs is rising rapidly. A recent population survey of 2,560 participants across Great Britain found that 2.9% reported current use of GLP-1 RAs to support weight loss, equating to approximately 1.6 million adults. Furthermore, approximately 3.3 million indicated they would consider pharmacological weight loss within the next year [14].

Preoperative GLP-1 RA use may reduce the risk of obesity related surgical complications. However, rapid weight loss may also increase the risk of malnutrition, which is itself associated with adverse outcomes, such as impaired wound healing and higher infection risk [15-17]. Johnson et al. identified malnutrition, defined by hypoalbuminaemia, as the strongest predictor of complications following total knee arthroplasty (TKA) in a cohort of 84,315 patients [18]. Thus, while targeted weight reduction may be beneficial, the balance between metabolic improvement and nutritional risks should also be addressed.

Early retrospective studies have begun examining outcomes of hip, knee and shoulder arthroplasty in patients receiving preoperative GLP-1 RAs, but findings remain inconsistent [19-33]. 

The aim of this systematic review is to evaluate the current evidence on preoperative GLP-1 RA use and postoperative outcomes following joint arthroplasty, and to highlight implications for future research and perioperative optimisation strategies.

## Review

Methods

A systematic search of PubMed, Embase, ClinicalTrials.gov and the Cochrane Library was conducted for articles published from inception to 29th July 2025, in accordance with the 2020 Preferred Reporting Items for Systematic Review and Meta-Analysis (PRISMA) guidelines [34]. A comprehensive search strategy implemented MeSH terms and keywords, combining terms ("joint replacement" OR "hip replacement" OR "knee replacement" OR "shoulder replacement" OR "arthroplasty"), ("semaglutide" OR "tirzepatide" OR "liraglutide" OR "GLP-1" OR "Glucagon-Like Peptide Receptor Agonist") and ("surgical outcomes" OR "complications" OR "recovery" OR "revision").

The search identified 112 articles. After removal of duplicates and incomplete records, 70 unique studies remained. Studies were included if they reported postoperative outcomes in adults undergoing arthroplasty with documented preoperative use of GLP-1 RAs. Exclusion criteria included case reports, reviews, meta-analyses, editorials, commentaries, and non-English publications.

Seventy titles and abstracts were independently reviewed by two authors (HT and IA), resulting in 41 studies for full-text assessment. Following full-text review, 15 studies met the inclusion criteria. Reference lists were screened to ensure no additional relevant studies were missed.

**Figure 1:**
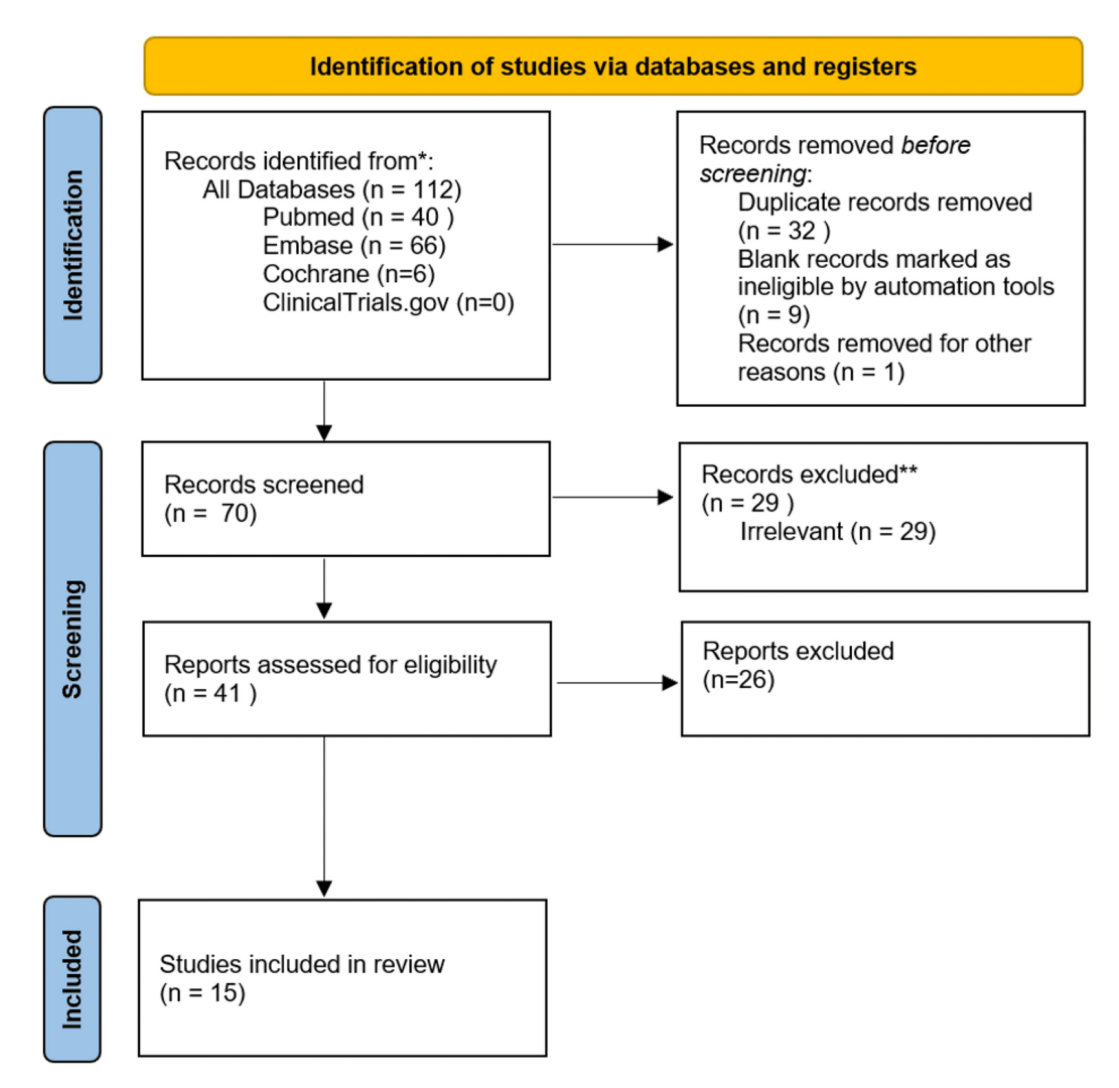
PRISMA flow diagram representing the study selection process PRISMA, Preferred Reporting Items for Systematic Reviews and Meta-Analyses.

Data extracted included study characteristics, population details, GLP-1 RA use, arthroplasty type, outcomes and follow-up. A PRISMA flow diagram illustrates the study selection process (Figure 1).

A meta-analysis was not conducted due to substantial heterogeneity between studies, including variation in patient populations, GLP-1 RA exposure definitions, arthroplasty type and outcome measures.

Study Characteristics

Fifteen studies met the inclusion criteria, comprising a total of 39,355 patients prescribed a GLP-1 RA prior to arthroplasty. Of these, seven studies assessed total hip arthroplasty (THA; 11,875 patients), six examined TKA (20,074 patients) and four evaluated total shoulder arthroplasty (TSA; 7,406 patients). Individual sample sizes ranged from 66 patients in the single-centre study by Magaldi et al. to 7,051 patients in the national database analysis by Magruder et al. [19,20]. All included studies were retrospective cohort designs conducted in the United States. Thirteen used national databases, whereas Magaldi et al. and Katzman et al. utilised patient data from a single institution [19,21]. Because multiple studies drew from overlapping large administrative datasets (e.g., PearlDiver (PearlDiver Technologies Inc., Colorado Springs, CO, USA); IBM MarketScan Commercial Claims and Encounters Database (IBM Corp., Armonk, NY, USA); TriNetX (TriNetX LLC, Cambridge, MA, USA), it is likely that some patients were included in more than one cohort, particularly for THA and TKA. The degree of overlap could not be quantified, but this raises the possibility that certain associations are emphasised by repeated analyses of the same underlying populations rather than independent replication. Buddhiraju et al. and Levidy et al. reported on outcomes for both TKA and THA within their cohorts [22,23].

Most studies evaluated 90-day postoperative complications, and the majority also reported one-year or two-year outcomes. Magaldi et al. was the only study to include patient-reported outcome measures [19]. Study characteristics for the THA, TKA and TSA cohorts are summarised in Tables 1-3.

**Table 1:** Summary of study characteristics for GLP-1 RA use in THA GLP-1 RA, glucagon-like peptide-1 receptor agonist; THA, total hip arthroplasty; T2DM, type 2 diabetes mellitus; OA, osteoarthritis. *PearlDiver (PearlDiver Technologies Inc., Colorado Springs, CO, USA); IBM MarketScan Commercial Claims and Encounters Database (IBM Corp., Armonk, NY, USA); TriNetX (TriNetX LLC, Cambridge, MA, USA)

Author (Year)	Journal	Sample size (Case), n	Sample size (Control), n	Patient data source*	Population details (age, sex, BMI, comorbidities)	GLP-1 RAs studied	Case group	Control group	Study period (years)	Matching strategy	Follow-up period
Buddhiraju et al. (2024) [22]	The Journal of Arthroplasty	1044	1044	TriNetX Database	Adult population aged 18 to 100 years undergoing THA	Not specified	Patients with a GLP-1 RA prescribed between 1 year and 15 days prior to surgery	Patients not prescribed GLP-1 RA	2005 to 2023	Propensity score matching (1:1) based on demographic variables and baseline comorbidities	90 days
Heo et al. (2024) [24]	The Journal of Arthroplasty	812	3248	IBM MarketScan commercial claims and encounters and Medicare supplemental and coordination of benefit databases	Adult population aged 18 and above with T2DM who underwent THA	Not specified	Patients with T2DM recorded as having at least three fills of their GLP-1 RA prescription, or if they received at least one fill of >90 days supply within six months prior to surgery	Patients with T2DM who did not use a GLP-1 RA within the six months prior to surgery	2016 to 2021	Propensity score matching (1:4) based on clinical characteristics	90 days, 1 year
Kim et al. (2024) [25]	The Journal of Arthroplasty	711 (90 day follow-up), 473 (2 year follow-up)	3084 (90 day follow-up), 1892 (2 year follow-up)	PearlDiver Database	Morbidly obese patients with a BMI ≥40 who had undergone a primary THA	Dulaglutide, exenatide, exenatide microsphere, liraglutide and semaglutide	Patients with BMI ≥ 40 who were on GLP-1 RA for at least 3 months pre and post-surgery	Patients with BMI ≥ 40 with no GLP-1 RA use	2010 to 2022	Propensity score matching (1:4) based on clinical characteristics	90 days, 2 years
Levidy et al. (2025) [23]	Journal of the American Academy of Orthopaedic Surgeons	2244	2244	TriNetX Database	All patients with T2DM undergoing THA	Dulaglutide, liraglutide, lixisenatide pramlintide, semaglutide and tirzepatide	T2DM Patients taking a GLP-1 RA within 1 year of surgery	T2DM Patients not taking a GLP-1 RA within 1 year of surgery	2005 to 2024	Propensity score matching (1:1) based on clinical characteristics	90 days, 1 year
Magaldi et al. (2024) [19]	Journal of the American Academy of Orthopaedic Surgeons	66	126	Single Institution	Patients between the ages of 18 and 89 who underwent a primary elective THA at a high-volume orthopaedic hospital	Dulaglutide, exenatide, liraglutide, and semaglutide	Patients with a GLP-1 RA included on their preoperative medication list	Patients without a GLP-1 RA included on their preoperative medication list	2016 to 2022	Covariate matching (1:2) based on age, sex and BMI	30 days, 12 weeks, 6 months and 1 year
Magruder et al. (2023) [26]	The Journal of Arthroplasty	1653	7812	PearlDiver Database	Patients with a diagnosis of DM, who underwent primary THA for OA	Semaglutide	Diabetic patients with an active prescription for semaglutide at the time of THA	Patients without a semaglutide prescription at the time of THA	2010 to 2021	Propensity score matching based on clinical characteristics	90 days, 2 years
Verhey et al. (2025) [27]	The Journal of Arthroplasty	5345	5345	PearlDiver Database	Adult patients without diabetes mellitus undergoing primary THA	Dulaglutide, exenatide, liraglutide, lixisenatide and semaglutide	Obese patients who were taking a GLP-1 RA between one year prior and 2 years after THA	Obese patients who were not taking a GLP-1 RA between one year prior and 2 years after THA	2010 to 2022	Propensity score matching (1:1) based on clinical characteristics	90 days, 2 years

**Table 2:** Summary of study characteristics for GLP-1 RA use in TKA GLP-1 RA, glucagon-like peptide-1 receptor agonist; OA, osteoarthritis; T2DM, type 2 diabetes mellitus; TKA, total knee arthroplasty. *PearlDiver (PearlDiver Technologies Inc., Colorado Springs, CO, USA); IBM MarketScan Commercial Claims and Encounters Database (IBM Corp., Armonk, NY, USA); TriNetX (TriNetX LLC, Cambridge, MA, USA).

Author (Year)	Journal	Sample size (Case), n	Sample size (Control), n	Patient data source*	Population details (age, sex, BMI, comorbidities)	GLP-1 RAs studied	Case group	Control group	Study period (Years)	Matching strategy	Follow-up period
Buddhiraju et al. (2024) [22]	The Journal of Arthroplasty	2095	2095	TriNetX Database	Adult population aged 18 to 100 years undergoing TKA	Not specified	Patients with a GLP-1 RA prescribed between 1 year and 15 days prior to surgery	Patients not prescribed GLP-1 RA	2005 to 2023	Propensity score matching (1:1) based on demographic variables and baseline comorbidities	90 days
Heo et al. (2024) [28]	Arthroplasty today	2388	2388	IBM MarketScan commercial claims and encounters and Medicare supplemental and coordination of benefit databases	Adult population aged 18 and above with T2DM who underwent TKA	Not specified	Patients with T2DM recorded as having at least three fills of their GLP-1 RA prescription, or if they received at least one fill of >90 days supply within six months prior to surgery	Patients with T2DM who did not use a GLP-1 RA within the six months prior to surgery	2016 to 2021	Propensity score matching (1:1) based on clinical characteristics	90 days, 1 year
Katzman et al. (2025) [21]	The Journal of Arthroplasty	865	8650	Single Institution	Adult patients (>18 years) who had a primary elective TKA with a minimum follow-up period of 90 days.	Albiglutide, dulaglutide, exenatide, liraglutide, lixisenatide, semaglutide and tirzepatide	Adult patients who used a GLP-1 RA within 6 months prior to surgery and continued use during 3 months after surgery	Adult patients who did not use GLP-1 RA	2012 to 2023	Propensity score matching (10:1) using a balanced, nearest-neighbour propensity score.	90 days, 2+ years
Kim et al. (2024) [29]	The Journal of Bone and Joint Society	2975 (90 day follow-up) and 1766 (2 year follow-up)	2975 (90 day follow-up) and 1766 (2 year follow-up)	PearlDiver Database	Morbidly obese patients with a BMI ≥40 who had undergone a primary elective unilateral TKA	Dulaglutide, exenatide, exenatide microspheres, liraglutide and semaglutide	Patients with BMI ≥ 40 who were prescribed a GLP-1 RA for at least 3 months prior to and after surgery	Patients with BMI ≥ 40 without GLP-1 RA use	2010 to 2022	Propensity score matching (1:1) based on clinical characteristics	90 days, 2 years
Levidy et al. (2025) [23]	Journal of the American Academy of Orthopaedic Surgeons	4700	4700	TriNetX Database	All patients with T2DM undergoing TKA	Dulaglutide, liraglutide, lixisenatide, pramlintide, semaglutide and tirzepatide	T2DM Patients taking a GLP-1 RA within 1 year of surgery	T2DM Patients not taking a GLP-1 RA within 1 year of surgery	2005 to 2024	Propensity score matching (1:1) based on clinical characteristics	90 days, 1 year
Magruder et al. (2023) [20]	The Journal of Arthroplasty	7051	34,524	PearlDiver Database	Patients with a diagnosis of DM who had a primary TKA for OA	Semaglutide	Diabetic patients with a prescription for semaglutide at the time of TKA	Patients not taking semaglutide	2010 to 2021	Propensity score matching (1:5) based on clinical characteristics	90 days, 2 years

**Table 3:** Summary of study characteristics for GLP-1 RA use in TSA GLP-1 RA, glucagon-like peptide-1 receptor agonist; ECI, Elixhauser comorbidity index; T2DM, type 2 diabetes mellitus; TSA, Total shoulder arthroplasty. *PearlDiver (PearlDiver Technologies Inc., Colorado Springs, CO, USA); IBM MarketScan Commercial Claims and Encounters Database (IBM Corp., Armonk, NY, USA); TriNetX (TriNetX LLC, Cambridge, MA, USA).

Author (Year)	Journal	Sample size (Case), n	Sample size (Control), n	Patient data source*	Population details (age, sex, BMI, comorbidities)	GLP-1 RAs studied	Case group	Control group	Study period (Years)	Matching strategy	Follow-up period
Choudhury et al. (2025) [30]	Journal of Shoulder and Elbow Surgery	505	7749	TriNetX Database	Adult patients with T2DM who underwent primary TSA	Albiglutide, dulaglutide, exenatide, liraglutide, lixisenatide, semaglutide and tirzepatide	Adult patients with T2DM with GLP-1 RA prescription	Adult patients with T2DM without GLP-1 RA prescription	2018 to 2023	None	90 days, 2 years
Elsabbagh et al. (2025) [31]	Journal of Shoulder and Elbow Surgery	5010 (90 day follow-up) and 3444 (2 year follow-up)	18,701 (90 day follow-up) and 12,692 (2 year follow-up)	PearlDiver Database	Adult population aged 18 and above with T2DM who underwent TSA	Dulaglutide, exenatide, liraglutide, lixisenatide and semaglutide	Patients with T2DM prescribed GLP-1 RA at the time of surgery	Patients with T2DM not on GLP-1 RA at the time of surgery	2010 to 2022	Propensity score matching (1:4) based on clinical characteristics	90 days, 2 year
Lawand et al. (2024) [32]	Journal of Shoulder and Elbow Surgery	1259 (90 day follow-up) and 766 (2 year follow-up)	1259 (90 day follow-up) and 766 (2 year follow-up)	TriNetX Database	Adults (>18 years) undergoing TSA, with all indications for TSA included and no exclusions	Liraglutide and semaglutide	Patients who had used a GLP-1 RA within 3 months before surgery	Patients who had not used GLP-1 RA within 3 months before surgery	2012 to 2023	Propensity score matching (1:1) based on clinical characteristics	90 days, 2 years
Seddio et al. (2024) [33]	Journal of Shoulder and Elbow Surgery	632	2302	PearlDiver Database	Adult population aged 18 and above with T2DM who underwent TSA for first time	Semaglutide	T2DM patients who used semaglutide within 1 year before TSA	T2DM patients who did not use semaglutide	2010 to 2022	Matched 1:4 age, Sex, ECI, Obesity, insulin, metformin, tobacco, end-organ diabetes complications	90 days

Risk of Bias Assessment

**Figure 2:**
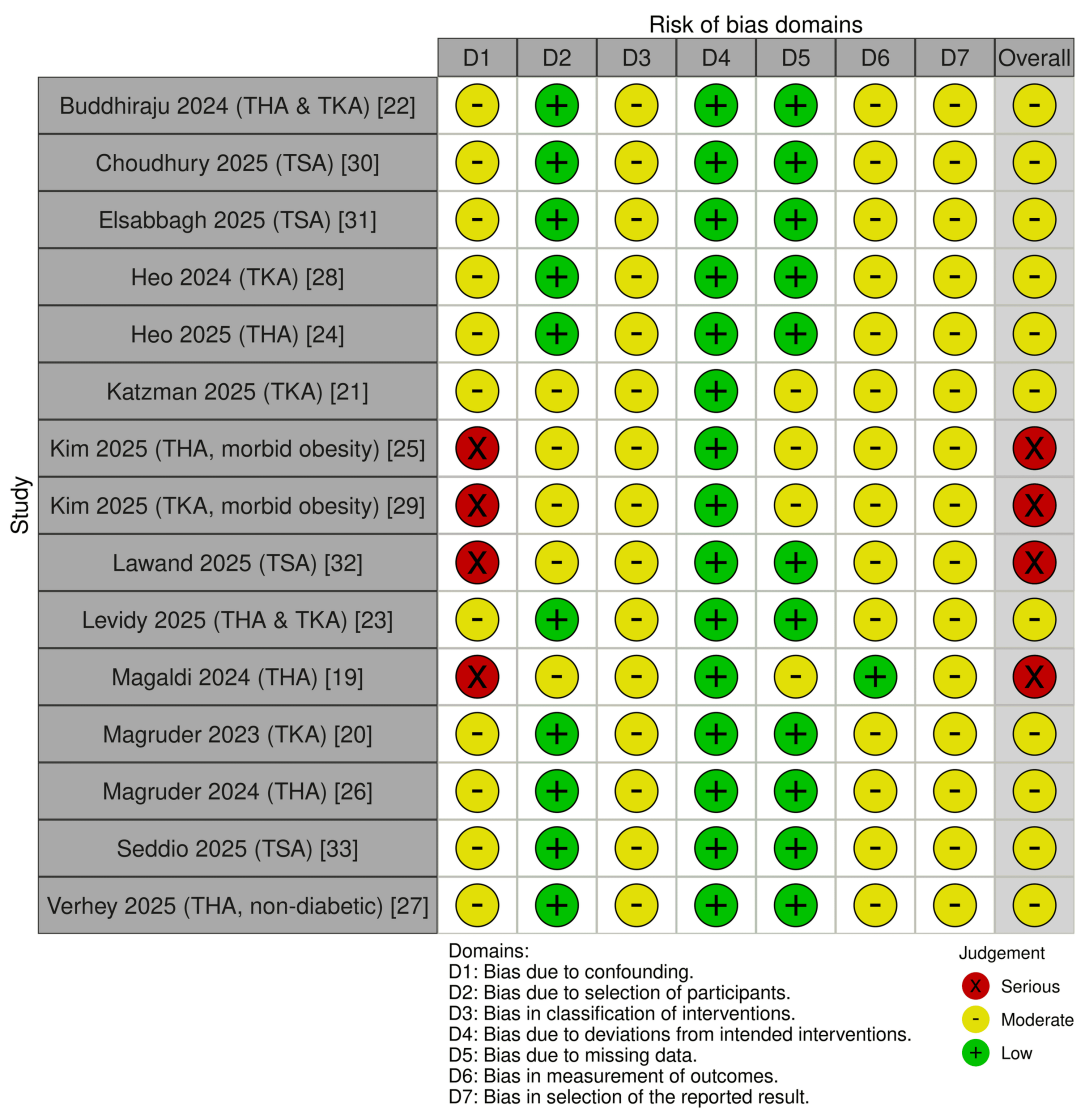
ROBINS-I traffic light plot Traffic light summary of ROBINS-I risk-of-bias assessments for each included study by domain and overall risk of bias [35]. ROBINS-I, Risk Of Bias In Non-randomized Studies - of Interventions.

**Figure 3:**
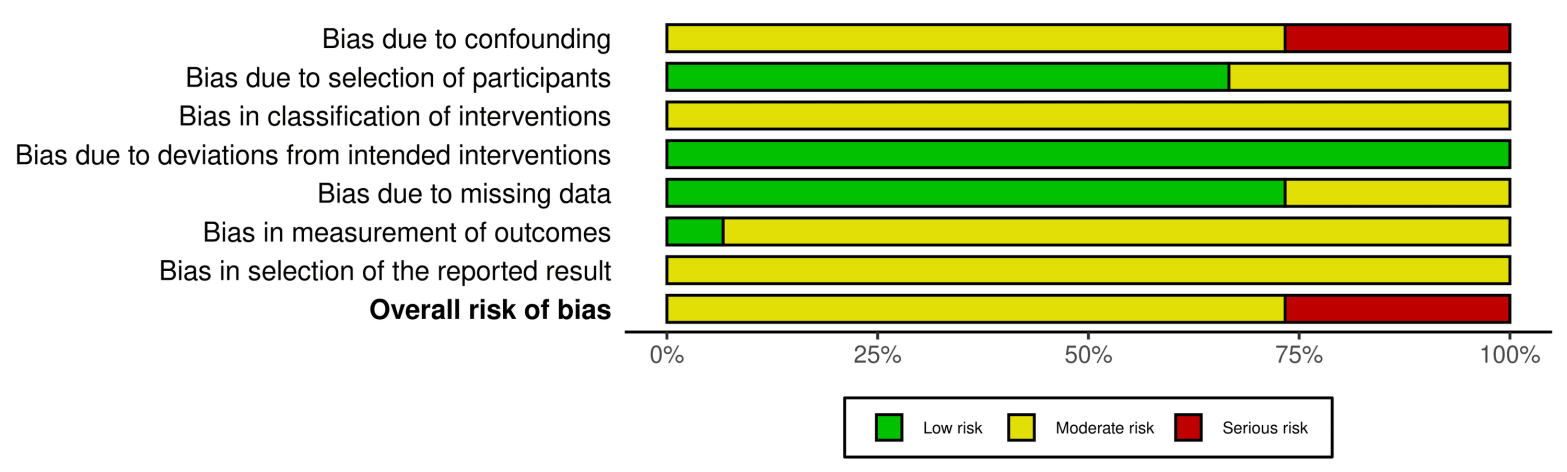
Bar chart summary of ROBINS-I domain-level risk of bias Bar chart demonstrating unweighted distribution of ROBINS-I domain-level risk of bias judgements across all included studies [35]. ROBINS-I, Risk Of Bias In Non-randomized Studies - of Interventions.

Studies included in this systematic review are observational, retrospective cohort analyses. The Risk Of Bias In Non-randomized Studies - of Interventions (ROBINS-I) tool for risk of bias assessment was used, with results summarised in Figures 2, 3 [35].

This is primarily due to residual confounding and the use of administrative or registry-based outcome coding. Although most studies utilised propensity score matching or multivariable adjustment, important confounders such as nutritional status, baseline glycaemic control, adherence to GLP-1 RA therapy, magnitude and rate of weight change on therapy and perioperative optimisation practices were not adequately accounted for. Four studies were rated at serious risk of bias due to confounding by indication and limited covariate adjustment. Other domains, including intervention classification and outcome measurement, were generally rated as moderate risk, while selection bias was low in large database studies. No study achieved an overall low risk of bias. Collectively, these findings indicate that the evidence base should be interpreted with moderate to low certainty. Because the studies were non-randomised and had at least moderate overall risk of bias, the certainty of the evidence according to contemporary grading frameworks would be considered low even where confidence intervals exclude the null.

Results

Total Hip Arthroplasty (THA)

Seven studies evaluated postoperative outcomes of GLP-1 RA use in patients undergoing THA (Table 4). 

**Table 4:** Summary of key findings for GLP-1 RA use in THA GLP-1 RA, glucagon-like peptide-1 receptor agonist; PJI, periprosthetic joint infection; ED, emergency department; PE, pulmonary embolism; DVT, deep vein thrombosis; SSI, surgical site infection; UTI, urinary tract infection; AKI, acute kidney injury; LOS, length of stay; VTE, venous thromboembolism; MI, myocardial infarction; THA, total hip arthroplasty.

Author (Year)	Outcomes reported	Key findings
Buddhiraju et al. (2024) [22]	PJI, readmission, revision, ED utilisation, superficial infection, deep infection, PE, DVT, aspiration, acute renal failure, mortality.	The cohort with prior use of GLP-1 RAs demonstrated a 42% significant reduction in the risk of PJI within 90 days (2.1% versus 3.6%; RR 0.58; P=0.042). There were no significant differences in risk of revision, PE, acute renal failure, readmission, ED utilisation or mortality.
Heo et al. (2024) [24]	90 days: SSI, PJI, wound dehiscence, periprosthetic fracture, cardiac arrest, stroke, pneumonia, DVT, UTI, AKI, c.diff infection, hypoglycaemic events, readmission, extended LOS (≥3 days). 1 year: revision, aseptic revision, PJI, periprosthetic fracture.	There were no statistically significant variations in 90-day surgical or medical complications, readmissions, or 1-year revision. However, the control cohort (not prescribed GLP-1 RAs) had a higher rate of extended hospital length of stay (28.5% versus 24.4%; OR 1.25; P=0.01])
Kim et al. (2024) [25]	90 days: readmission, any medical complication, AKI, cardiac arrest, DVT, wound dehiscence, hematoma, nerve injury, pneumonia, PE, transfusion, UTI, all cause revision, PJI, periprosthetic fracture, aseptic loosening. 2 years: all cause revision, PJI, periprosthetic loosening.	The use of GLP-1 RAs in morbidly obese patients prior to primary THA was associated with reduced rates of PJI (1.6% versus 3.2%; OR 0.5; P=0.03), readmission (6.9% versus 9.7%; OR: 0.7; P=0.04), any medical complication (10.5% versus 14.1%; OR: 0.7; P=0.03), and hematoma formation (0.0% versus 1.3%; OR: 0.1; P<0.01) at 90 days. At 2 years, there were no statistically significant differences in the rates of surgical complications for the two groups.
Levidy et al. (2025) [23]	PJI, revision, periprosthetic fracture.	No statistically significant differences were identified in rates of PJI, revision and periprosthetic fracture at 90 days nor at 1 year.
Magaldi et al. (2024) [19]	30 days: LOS, inpatient complications, postop pain levels, ED visits, readmissions, The Hip osteoarthritis outcome score for joint replacement (HOOS-JR) was completed preoperatively and at 12 weeks, 6months and 1 year.	Postoperative nausea and vomiting was higher in the GLP-1 RA cohort (18.2% versus 6.0%; P= .011), however, their use was not associated with any notable differences in the length of stay or readmission rates. Inpatient pain levels were reported as equivalent between the matched cohorts. No statistically significant differences were reported between these two groups when comparing the Hip Osteoarthritis Outcome Score for joint replacement.
Magruder et al. (2023) [26]	90 days: readmission, LOS, care costs, cerebrovascular accident, PE, DVT, VTE, MI, pneumonia, AKI, hypoglycaemic events, sepsis. 2 years: periprosthetic fracture, PJI, aseptic loosening, revision.	Patients taking semaglutide at the time of primary THA had lower 90-day readmission rates (6.2% versus 8.8%; OR 0.68; P= .0004) and lower rates of PJI within 2 years (1.6% versus 2.9%; OR 0.56; P= .005) compared to the control cohort. There were no notable differences in medical complications, implant related complications, and length of stay between the two cohorts.
Verhey et al. (2025) [27]	90 days: DVT, PE, Anaemia, Transfusion, MI, TIA, Stroke, Aspiration pneumonia, AKI, sepsis, ED visit, readmission, mortality, wound dehiscence, hematoma, periprosthetic fracture, PJI, aseptic loosening, SSI, instability, revision. 2 years: periprosthetic fracture, PJI, aseptic loosening, SSI, instability, revision.	Obese non-diabetic patients taking GLP-1 RAs were less likely to visit the ED within 90 days (4.8% versus 5.8%; OR 0.81; P=0.020), had reduced acute blood loss anaemia (0.4% versus 0.7%; OR 0.57; P=0.043) and a reduced postoperative transfusion requirement (0.7% versus 1.4%; OR 0.53; P=0.001). Patients within the two cohorts had a comparable risk of developing acute ischaemic stroke, DVT, PE, MI, pneumonia, AKI and sepsis. There was also no notable difference in the risk of PJI, periprosthetic fracture, aseptic loosening or requiring revisions.

Three of these studies reported improved outcomes, especially reduced rates of PJI and 90-day readmission among GLP-1 RA users [22,25,26]. Improved outcomes were most frequently observed in cohorts with a higher prevalence of diabetes or morbid obesity [22,24-26]. Several studies also noted fewer medical complications and shorter in-patient length of stay [24,27]. Levidy et al. found no significant differences in outcomes, and Magaldi et al. reported increased postoperative nausea among GLP-1 RA users [19,23]. Overall, several THA studies reported more favourable outcomes among GLP-1 RA users, particularly for PJI and readmission, whereas others found no differences or identified specific adverse effects such as increased postoperative nausea; effects were not consistently observed across all cohorts.

Total Knee Arthroplasty (TKA)

Six studies evaluated the postoperative outcomes of GLP-1 RA use in patients undergoing TKA (Table 5).

**Table 5:** Summary of key findings for GLP-1 RA use in TKA GLP-1 RA, glucagon-like peptide-1 receptor agonist; PJI, periprosthetic joint infection; ED, emergency department; PE, pulmonary embolism; DVT, deep vein thrombosis; SSI, surgical site infection; UTI, urinary tract infection; AKI, acute kidney injury; LOS, length of stay; T2DM, type 2 diabetes mellitus; TKA, total knee arthroplasty; VTE, venous thromboembolism; MI, myocardial infarction.

Author (Year)	Outcomes reported	Key findings
Buddhiraju et al. (2024) [22]	90-day readmission, revision, ED utilisation, superficial infection, deep infection, PE, DVT, aspiration, acute renal failure, mortality.	Use of GLP-1 RA preoperatively was associated with a 47% reduced risk of readmission within 90 days (1.1% versus 2.0%, RR=0.53, P=0.017), with no notable differences in the remaining outcomes evaluated.
Heo et al. (2024) [28]	90 days: SSI, PJI, wound dehiscence, periprosthetic fracture, cardiac arrest, stroke, pneumonia, DVT, UTI, AKI, Clostridium difficile infection, hypoglycaemic events, readmission, extended LOS (≥3 days). 1 year: revision, aseptic revision, PJI, periprosthetic fracture.	GLP-1 RA use in T2DM patients was associated with shorter lengths of hospital stay (25.4% versus 31.2%; OR 1.29; P<0.001), with no significant differences observed in the other outcomes assessed.
Katzman et al. (2025) [21]	LOS, Discharge disposition, 90- day ED visits, 90-day readmissions. 2-year and any-time revision rates. BMI trends over time (5-year pre-op to 5-year post-op) and same-day TKA cancellations.	Patients using GLP-1RAs experienced a higher risk of ED visits within 90 days post-operation (5.9% versus 4.0%, P=0.008); however, there was no notable difference in readmission within 90 days (4.3% versus 3.6%; P=0.168). Anytime revision risk was lower (2.7% versus 3.9%; P=0.034), but not statistically significant at two years (2.3% versus 2.6%; P=0.362).
Kim et al. (2024) [29]	90 days: readmission, any medical complication, AKI, cardiac arrest, DVT, wound dehiscence, hematoma, nerve injury, pneumonia, PE, Transfusion, UTI, all cause revision, PJI, periprosthetic fracture, aseptic loosening. 2 year: all cause revision, PJI, periprosthetic loosening.	Morbidly obese who were on GLP-1 RAs had significantly reduced rates at 90 days of PJI (1.0% versus 1.8%; P=0.037), medical complications (10.6% versus 12.7%; P=0.033), PE (<0.4% versus 0.6%; P=0.05) and readmissions (5.3% versus 8.9%, P<0.001), with no significant differences in all-cause revision, periprosthetic fracture and aseptic loosening rates. Interestingly, Kim et al compared the outcomes for the group of morbidly obese patients using GLP-1 RAs with a group of patients with severe obesity and found they yielded similar complication rates.
Levidy et al. (2025) [23]	PJI, revision, periprosthetic fracture.	There were notably lower rates of PJI in T2DM patients using a GLP-1 RAs at 3 months (0.94% versus 1.45%; P<0.001) and 1 year (1.21% versus 2.04%; P <0.001). However, rates of periprosthetic fractures were notably increased for the GLP-1 RA therapy group at both 3 months (0.47% versus 0.21%; P=0.034) and 1 year (0.70% versus 0.34%; P=0.015). There was no notable difference in revisions.
Magruder et al. (2023) [20]	90 days: readmission, LOS, care costs, cerebrovascular accident, PE, DVT, VTE, MI, pneumonia, AKI, hypoglycaemic events, sepsis. 2 years: periprosthetic fracture, PJI, aseptic loosening, revision.	Diabetic semaglutide users had a higher incidence of AKI (4.9% versus 3.9%; OR 1.28; P<0.001), pneumonia (2.8% versus 1.7%; OR 1.67; P<0.001), and MI (1.0% versus 0.7%; OR 1.49; P=0.003) within 90 days of TKA. Conversely the semaglutide group had a significant decrease in the risk of developing sepsis (0.0% versus 0.4%; OR 0.23; P<0.001), readmission (7.0% versus 9.4%; OR 0.71; P<0.001), PJI at 2 years (2.1% versus 3.0%; OR 0.7; P<0.001) and 2-year revision (4.0% versus 4.5%; OR 0.86; P=0.02).

Three studies reported exclusively favourable outcomes: Buddhiraju et al. observed reduced risk of readmission, Heo et al. reported a shorter length of hospital stay and Kim et al. found reductions in PJI, any medical complications and readmission within 90 days [22,28,29]. However, other studies results were mixed: Levidy et al. noted higher rates of periprosthetic fracture among GLP-1 RA users at both 90 days and one year, despite lower PJI risk over the same period [23]. Katzman et al. observed more ED visits within 90 days but fewer any time revisions [21]. Magruder et al. reported increased odds of myocardial infection (MI), acute kidney injury (AKI) and pneumonia but reduced risks of sepsis, readmission, PJI and revision in semaglutide users [20]. Overall, GLP-1 RA use prior to TKA was associated with a mix of potentially favourable and unfavourable short-term outcomes, and findings across studies remained heterogenous and partially conflicting.

Total Shoulder Arthroplasty (TSA)

Four studies assessed GLP-1 RA use in patients undergoing TSA (Table 6).

**Table 6:** Summary of key findings for GLP-1 RA use in TSA GLP-1 RA, glucagon-like peptide-1 receptor agonist; PJI, periprosthetic joint infection; ED, emergency department; PE, pulmonary embolism; DVT, deep vein thrombosis; SSI, surgical site infection; UTI, urinary tract infection; AKI, acute kidney injury; LOS, length of stay; T2DM, type 2 diabetes mellitus; BMI, body mass index; TKA, total knee arthroplasty; VTE, venous thromboembolism; MI; myocardial infarction.

Author (Year)	Outcomes reported	Key findings
Choudhury et al. (2025) [30]	90 days: cerebrovascular accident, MI, PE, DVT, AKI, sepsis, UTI, dehiscence, SSI, respiratory complications, coma, stroke, mortality, readmission, ED visits. 2 years: periprosthetic fracture, PJI, aseptic loosening, revision.	GLP-1 RA use appears to be associated with reduced odds of postoperative 90-day mortality in T2DM (OR 0.17, 95% CI 0.0043-0.99, P=0.0435), with no significant differences observed in the other outcomes assessed.
Elsabbagh et al. (2025) [31]	90 days: LOS, DVT, cardiac arrest, MI, cerebrovascular accident, pneumonia, UTI, SSI, hypoglycaemic event, sepsis, readmission. 2 years: postoperative all-cause revision, PJI, PPF and aseptic revision.	The diabetic patients taking GLP-1 RAs had no notable difference in any of the outcomes studied.
Lawand et al. (2024) [32]	90 days: PE, DVT, MI, anaemia, pneumonia, renal failure, blood transfusion, readmission. 2 years: periprosthetic fractures, PJI, mechanical loosening revision.	The GLP-1 RA cohort had higher rates of DVT (1.6% versus 0.9%; OR 3.0; P=0.001), transfusion (7.1% versus 4.3%; OR 1.7; P=0.003), pneumonia (3.3% versus 1.5%; OR 2.3; P=0.003), MI (1.6% versus 0.9%; OR 2.8; P=0.003) and readmission (8.1% versus 5.2%; OR 1.6; P=0.004) compared to control cohort.
Seddio et al. (2024) [33]	SSI, Cardiac events, VTE, sepsis, prosthetic dislocation, Pneumonia, UTI, wound complication, AKI, ED visit, readmission.	T2DM patients using semaglutide within 1 year before TSA had significantly reduced adverse events within 90 days compared to matched controls (18.8% versus 41.9%; P<0.001), including lower odds of SSI (<1.7% versus 3.0%; P=0.002), VTE (1.9% versus 5.0%; P=0.001), pneumonia (3.0% versus 10.4%, P<0.001) and ED attendance (26.6% versus 46.7%; P<0.001).

Two studies reported favourable outcomes: Choudhury et al. demonstrated reduced 90-day mortality and Seddio et al. observed fewer adverse events including surgical site infection (SSI), venous thromboembolism (VTE), pneumonia and ED attendance [30,33]. One study found no notable differences in perioperative complications [31]. Lawand et al. included all indications for arthroplasty and reported higher rates of deep vein thrombosis (DVT), MI, pneumonia, transfusion and readmission in GLP-1 RA users [32]. Collectively, the evidence for TSA currently presents a mixed picture. 

Discussion

In recent years, the use of GLP-1 RAs has expanded, moving from predominantly glycaemic control in diabetes to becoming a powerful pharmacological weight loss therapy [36]. As increasing numbers of adults initiate GLP-1 RAs for weight loss, more arthroplasty patients will be using these medications preoperatively. Yet their impact on postoperative outcomes remains uncertain due to limited, heterogeneous evidence.

Across arthroplasty types, approximately two-thirds of studies reported favourable postoperative outcomes among GLP-1 RA users, primarily reduced PJI and readmission rates after THA and TKA. However, discrepancies exist: Buddhiraju et al. found PJI reduction in THA but not TKA, while Kim et al. reported reductions in both and Levidy et al. found benefit for TKA but not THA [22,23,25,29]. These inconsistencies may reflect procedural differences - THA’s simpler biomechanics versus TKA’s greater soft-tissue disruption - or patient selection, as THA studies had higher diabetic proportions. TSA evidence was sparse and contradictory: Choudhury et al. reported reduced 90-day mortality, Seddio et al. found fewer adverse events, while Lawand et al. observed higher complication rates, likely due to inclusion of all arthroplasty indications rather than primary elective procedures [30,32,33]. Improved outcomes were most consistent in diabetic and morbidly obese patients, but inconsistent in non-diabetic or non-obese cohorts, where evidence remains sparse. Clinically, these findings suggest GLP-1 RAs may aid preoperative optimisation for high-risk THA/TKA candidates, though confirmation requires prospective studies, particularly in non-obese and non-diabetic cohorts. Careful nutritional and metabolic assessment is advisable for preoperative GLP-1 RA users, particularly with rapid weight loss, to balance metabolic benefits against malnutrition risks.

Direct comparison of outcomes across studies is limited by considerable heterogeneity across multiple domains. Patient populations varied widely: some cohorts were restricted to T2DM patients, others included all adults regardless of diabetic status, several assessed morbidly obese patients, and only one evaluated non-diabetic obese patients [27]. GLP-1 RA exposure definitions differed substantially: some classified exposure based on a single prescription fill within a broad preoperative window, others required multiple fills or prolonged treatment, and only a few required continued therapy postoperatively. These likely correspond to varying degrees of weight loss and metabolic control, precluding determination of whether outcomes reflect brief preoperative exposure, sustained therapy, or postoperative management. GLP-1 RAs also varied from older drugs (exenatide, liraglutide) to newer long-acting analogues (semaglutide, tirzepatide), with differing efficacy, pharmacokinetics and side-effect profiles [37]. This heterogeneity limited the feasibility of any formal quantitative synthesis and precluded determination of whether apparent benefits or complications are attributable to the drug itself, underlying patient factors, or selection bias.

Follow-up duration was limited across studies (≤two years), leaving longer-term effects unknown. Only Magaldi et al. assessed patient-reported outcomes, identifying increased postoperative nausea and vomiting among GLP-1 RA users but no other significant differences [19]. More comprehensive patient-reported outcome data and longer-term follow-up are essential to determine whether short-term differences translate into improved function, recovery or reduced revision risk.

The mechanisms by which GLP-1 RAs may influence surgical outcomes remain hypothetical but could be multifactorial. Weight loss and improved glycaemic control directly address two major PJI risk factors - obesity drives chronic inflammation and impairs immune function, while hyperglycaemia promotes bacterial growth and biofilm formation [38,39]. GLP-1 RAs also exert anti-inflammatory effects by reducing systemic markers such as C-reactive protein (CRP), tumour necrosis factor (TNF)-alpha and IL-6, and affect immune cell receptor signalling [40,41]. Lower BMI also facilitates technically easier procedures with reduced operative time and tissue trauma [42]. These pathways may explain observed reductions in PJI and readmissions, though direct confirmation requires prospective mechanistic studies. Conversely, gastrointestinal side-effects such as nausea and vomiting, potential nutritional deficits, and risk of sarcopenia from rapid weight loss without strength training could contribute to adverse outcomes in some contexts [19,43].

Future research should adopt standardised GLP-1 RA exposure definitions, explicitly report the timing and duration relative to surgery and continuation/cessation protocols. Studies should quantify preoperative weight loss amount/rate and relate these to outcomes, providing more meaningful insight than drug exposure alone. Comparative analyses between diabetic and non-diabetic GLP-1 RA users are needed to clarify metabolic profile effects, alongside risk-factor stratified analyses by diabetes status, BMI and weight loss magnitude, to distinguish drug effects from patient characteristics. International studies from diverse populations and healthcare settings are needed to establish generalisability beyond US practice. 

This review has several limitations. All included studies were retrospective analyses of US electronic health records or administrative databases, which are prone to coding errors and misclassification. The risk of bias assessment further highlights these methodological constraints: using the ROBINS-I tool, most studies were judged to have a moderate risk of bias and four were rated as having serious risk of bias, primarily due to residual confounding, incomplete adjustment for key variables and reliance on registry-based coding [35]. No study achieved an overall low risk of bias. These limitations reduce the certainty of the observed associations and may contribute to the inconsistencies across arthroplasty types and patient sub-groups. The absence of prospective cohort studies or randomised controlled trials limits causal inference and robust control of confounding, even with matching or statistical adjustment. Furthermore, several analyses drew on the same national or commercial databases, and unique patients could not be identified across studies, so apparent consistency may partly reflect re-analyses of overlapping cohorts, potentially inflating the perceived weight of evidence. Unmeasured confounders such as glycaemic control, perioperative weight change, nutritional status and treatment adherence precluded quantitative synthesis, and very few studies reported detailed data on these factors, which are biologically linked to both GLP-1 RA prescribing and postoperative outcomes. Residual confounding is therefore highly likely, even in propensity-matched analyses. Given this exclusively retrospective, non-randomised evidence base, the associations reported in this review should not be interpreted as demonstrating clinical benefit, but rather as hypotheses generating to be tested in future prospective studies, with clearly defined exposure windows, standardised outcome definitions, and appropriate adjustment for key confounders.

## Conclusions

For practising orthopaedic surgeons, current evidence does not yet support initiating or discontinuing GLP-1 RAs solely to modify arthroplasty outcomes. However, observational data suggest that preoperative GLP-1 RA use may be associated with improved short-term outcomes, particularly in diabetic and morbidly obese patients, with the most frequently reported benefits being reductions in PJI and hospital readmission after hip and knee arthroplasty. Results across studies remain heterogeneous, evidence for shoulder arthroplasty is limited and sometimes conflicting, and the exclusively retrospective designs, use of large administrative datasets, and incomplete adjustment for key confounders substantially restrict the strength of any causal interpretation. Perioperative decision making should therefore continue to prioritise established optimisation measures - smoking cessation, glycaemic control, nutritional assessment, and weight management - while GLP-1 RA prescribing is individualised in collaboration with general practice, endocrinology and anaesthesia teams.

Current data are reassuring that continued use in appropriately selected high-risk patients does not appear to be associated with large increases in perioperative complications. Future prospective cohort studies and randomised controlled trials are needed to clarify the mechanisms underlying the observed associations, assess longer-term outcomes and determine whether effects differ by diabetic status, degree and rate of weight loss, perioperative cessation protocols or specific GLP-1 RAs. Until such evidence is available, clinicians should interpret existing findings with caution, but may reasonably regard GLP-1 RAs as a promising component of a broader, multidisciplinary preoperative optimisation strategy in carefully selected high-risk arthroplasty candidates.
